# Using real-world evidence data and digital monitoring to analyze the hepatotoxic profiles of biologics across more than two million patients

**DOI:** 10.1038/s41598-023-37979-0

**Published:** 2023-07-05

**Authors:** Priyanka Banerjee, Saskia Preissner, Robert Preissner

**Affiliations:** 1 Institute for Physiology and Science-IT, Charite, University Medicine Berlin, 10115 Berlin, Germany; 2 Department Oral and Maxillofacial Surgery, Berlin Institute of Health, Charité-Universitätsmedizin Berlin, Corporate Member of Freie Universität Berlin, Humboldt-Universität zu Berlin, Berlin, Germany

**Keywords:** Drug discovery, Rheumatology, Risk factors

## Abstract

The real-world evidence data from multiple sources which includes information on patient health status and medical behavior in routine clinical setup can give deeper insights into drugs ‘safety and efficacy. The RWE-based analysis in this study revealed a statistically significant link between biologics usage and hepatotoxicity in patients. To the best of our knowledge, this study is the first to conduct a large-scale multi-cohort analysis on the hepatotoxic profiles of biologics. Biologics are among the most prescribed medicines for several chronic inflammatory diseases. These agents target critical pathogenic pathways, but they may also have serious side effects. It is important to analyze whether biologics agents are an added concern or therapeutic opportunity. Real-world evidence (RWE) data were extracted for patients using biologics to monitor the safety and effectiveness of the biologics. All six biologics included in this analysis—are mostly highly prescribed biologics. The aim of the study was to assess the hepatotoxic profiles of subjects using different biologics. We evaluated the safety of current treatment regimens for patients in a large real-world cohort from multiple health care centers. Total number of eligible patients retrieved from the database is 38,112,285. Of these 38 million patients, 2.3 million take biologics. The primary objective was to assess the potential adverse hepatotoxic effects of the six biologics; adalimumab, trastuzumab, prevnar13, pegfilgrastim, interferon-beta1a and insulin glargine across different indications like diabetes mellitus, encounter for immunization, malignant neoplasm of breast, multiple sclerosis, malignant neoplasm of kidney, aplastic anaemias, radiation sickness, Crohn's disease, psoriasis, rheumatoid arthritis, spondylopathies. Data from patients using the six most-used biologics-adalimumab, trastuzumab, prevnar13, pegfilgrastim, interferon-beta1a and insulin glargine were retrieved from a global research network covering 250 million patients’ data from 19 countries, and assigned to the cohorts 1 and 2, respectively. The cohorts were propensity score matched for age and sex. After defining the primary outcome as “hepatotoxicity” (endpoint defined as ICD-10 code: K71 (hepatotoxic liver disease), a Kaplan–Meier survival analysis was performed, and risk ratios (RR), odds ratios (OR), and hazard ratios (HR) were determined. A total number of 2,312,655 subjects were eligible who take biologics, and after matching total cohorts accounted for 2,303,445. We have considered the clinical data as a 1:1 matched‐study design, using propensity score‐matched sub‐cohorts to better control for confounding associations that might stem from different distributions of age and gender between the whole dataset and the subset of patients. We discovered evidence supporting the hepatotoxic-causing effect of biologic drugs: (i) all biologics considered together had an OR of 1.9 (95% CI, 1.67–2.35), with (ii) Adalimumab 1.9 (95% CI, 1.72–2.20), Trastuzumab 1.7 (95% CI, 1.2–2.3), Prevnar13 2.3 (95% CI, 2.16–2.60), Pegfilgrastim 2.3 (95% CI, 2.0–2.50), Interferon-Beta1a 1.7 (95% CI, 1.18–2.51), and Insulin glargine 1.9 (95% CI, 1.8–1.99). Our findings indicate that clinicians should consider evaluating hepatic profiles of patients undergoing treatment with biologic drugs and counsel them regarding the risk of developing hepatic injury. Strengths of the study includes a large sample size and robust statistical techniques. Limitations of this study include lack of detailed information regarding clinical severity. Major biologics are associated with hepatotoxicity. We discovered evidence supporting the hepatotoxicity-causing effects of biologics: all biologics considered together had an OR of 1.9 (95% CI, 1.67–2.35).

## Introduction

Biologics are among the most prescribed medicines for several chronic inflammatory diseases. These agents target critical pathogenic pathways, but they may also have serious side effects^1^. It is important to analyze whether biologics are an added concern or therapeutic opportunity^1,2^. Real-world evidence (RWE) relies on data that got collected outside of the controlled setting of a clinical trial to assess the safety and effectiveness of a medication. Using these real-world data (RWD) it is possible to cover the clinical insights on potential benefits or risks of medicine. Most of the information is derived from registries, EHRs, insurance claims and billing data as well as mobile devices used by the patients^3^. Clinical trials are important as they gather information on the medication’s performance patient by patient, data point by data point and evaluate their effects on human health outcomes. However, besides the data from clinical studies, data on RWE is critically important for evaluating biologics' safety and effectiveness^4^. Several studies have been published supporting the adverse effects of biologics. However these studies were limited to number of patients and there were no consistency across the outcomes as a result caution is needed interpreting these studies’ outcomes^5^. There is certainly a need for more research regarding the long-term safety of biologics and a comparative safety analysis of different biologics, preferably without industry involvements.

The present study aimed at addressing the reality of hepatotoxic side-effects associated with the usage of biologics—by investigating large real-world cohorts from multiple health care centers. For this intent, the TriNetX Global Health Research Network (TriNetX, Cambridge, Massachusetts, USA, https://trinetx.com/) was used to gain related retrospective data, as it provides access to a significant number of medical records^6^. TriNetX is a database is a platform that administers clinical patients data from more than 120 health care organizations (HCOs) from 19 countries. By May 2022, TriNetX had collected digital clinical records from more than 250 million subjects. The network is well established to research on medical topics of worldwide interest, including the coronavirus disease 2019 (COVID-19) pandemic^4^.

## Methods

### Ethical approval

TriNetX is compliant with the Health Insurance Portability and Accountability Act (HIPAA), the US federal law which protects the privacy and security of healthcare data^6^. TriNetX is certified to the ISO 27001:2013 standard and maintains an Information Security Management System (ISMS) to ensure the protection of the healthcare data it has access to and to meet the requirements of the HIPAA Security Rule. Any data displayed on the TriNetX Platform in aggregate form, or any patient-level data provided in a data set generated by the TriNetX Platform, only contains de-identified data as per the de-identification standard defined in Section §164.514(a) of the HIPAA Privacy Rule. The process by which the data is de-identified is attested to through a formal determination by a qualified expert as defined in Section §164.514(b)^1^ of the HIPAA Privacy Rule. This formal determination by a qualified expert refreshed in December 2020, supersedes the need for TriNetX’s previous waiver from the Western Institutional Review Board (IRB). The TriNetX network contains data provided by participating Healthcare Organizations (HCOs), each of which represents and warrants that it has all necessary rights, consents, approvals and authority to provide the data to TriNetX under a Business Associate Agreement (BAA), so long as their name remains anonymous as a data source and their data are utilized for research purposes. The data shared through the TriNetX Platform are attenuated to ensure that they do not include sufficient information to facilitate the determination of which HCO contributed with specific information about a patient^12^.

### Data acquisition, inclusion and exclusion criteria

The TriNetX database was accessed on May, 2022. The time window was defined as five years after meeting the index event. Furthermore, the primary outcome was defined as “hepatotoxic”, whereby a daily time interval was used to record outcome events. The eligibility period was limited to 20 years back from the index event. Additionally, the the survival rate of patients treated with biologics or without biologics was compared.

### Matching process

All cohorts were stratified and balanced via propensity matching for age at index event and gender distribution to mitigate confounder bias. In order to replicate randomized conditions as closely as possible one-to-one matching was applied. The additional cohorts consisting of patients treated with respective or without respective biologics -were analogously matched with cohort 1 and cohort 2 for the respective analyses (for each biologic).

### Data analysis

Kaplan–Meier survival analyses were performed, and risk ratios (RR), odds ratios (OR), as well as hazard ratios (HR) were determined for the studied cohorts. Statistical testing was conducted using the Log-Rank test, whereby the significance threshold was defined as p ≤ 0.05.

### Adalimumab—TNF-α inhibitor (monoclonal antibody)

Adalimumab is a monoclonal antibody to human tumor necrosis factor (TNF) alpha which is used for the treatment of rheumatoid and psoriatic arthritis^7^. Adalimumab has been linked to rare instances of idiosyncratic acute liver injury and is a potential cause of reactivation of hepatitis B^8^.

A total of 58 providers responded with patients. The final cohort1 included 124,110 patients who matched the query criteria. A total of 67 providers responded with patients and the final cohort2 included 7,831,122 patients who matched the query criteria. Propensity score matching was performed on all listed characteristics. Characteristics of the cohorts before and after matching are summarized in the supplementary Table S1.

### Trastuzumab-inhibits HER2 homodimerization

Trastuzumab is an anti-cancer drug used for targeted therapy, mainly used for the treatment of cancers that have large amounts of a protein called human epidermal growth factor receptor 2 (HER2)^9^. One of the most severe adverse events reported with Trastuzumab is hepatotoxicity.

A total of 53 providers responded with patients. The final cohort1 included 24,484 patients who matched the query criteria. A total of 67 providers responded with patients and the final cohort2 included 757,227 patients who matched the query criteria. Propensity score matching was performed on all listed characteristics. Characteristics of the cohorts before and after matching are summarized in the supplementary Table S2.

### Prevnar13-a vaccine

Prevenar 13 is a vaccine and it is available as injection that contains parts from 13 different types of the bacterium *Streptococcus pneumoniae* (*S. pneumoniae*)^10^.

A total of 41 providers responded with patients. The final cohort1 included 551,638 patients who matched the query criteria. A total of 61 providers responded with patients and the final cohort2 included 12,018,791 patients who matched the query criteria. Propensity score matching was performed on all listed characteristics. Characteristics of the cohorts before and after matching are summarized in the supplementary Table S3.

### Pegfilgrastim-pegylated granulocyte colony-stimulating factor

Pegfilgrastim is available as injection and is used to treat neutropenia. It is a pegylated granulocyte colony-stimulating factor that is FDA-approved for patients receiving cancer medicines^11^.

A total of 58 providers responded with patients. The final cohort1 included 130,096 patients who matched the query criteria. A total of 67 providers responded with patients and the final cohort2 included 9,742,731 patients who matched the query criteria. Propensity score matching was performed on all listed characteristics. Characteristics of the cohorts before and after matching are summarized in the supplementary Table S4.

### Interferon-beta1a-balances the expression of pro- and anti-inflammatory agents

Interferon beta-1a is an active ingredient from the group of interferons that is used to treat relapsing forms of multiple sclerosis. The medicine reduces the occurrence and severity of flare-ups and delays the progression of the disease^12^. Interferon beta is a well known cause of mild hepatic injury that occasionally can lead to severe liver injury with jaundice^8^.

A total of 54 providers responded with patients. The final cohort1 included 21,097 patients who matched the query criteria. A total of 67 providers responded with patients and the final cohort2 included 220,526 patients who matched the query criteria. Propensity score matching was performed on all listed characteristics. Characteristics of the cohorts before and after matching are summarized in the supplementary Table S5.

### Insulin glargine

Insulin glargine, is a long-acting modified form of medical insulin, used in the management of type I and type II diabetes^13^.

In this study, a total of 58 providers responded with patients. The final cohort1 included 1,466,230 patients who matched the query criteria. A total of 67 providers responded with patients. The final cohort2 included 5,0889,075 patients who matched the query criteria. Propensity score matching was performed on all listed characteristics. Characteristics of the cohorts before and after matching are summarized in the supplementary Table S6.

### Assessment, allocation and matching

**Figure1:**
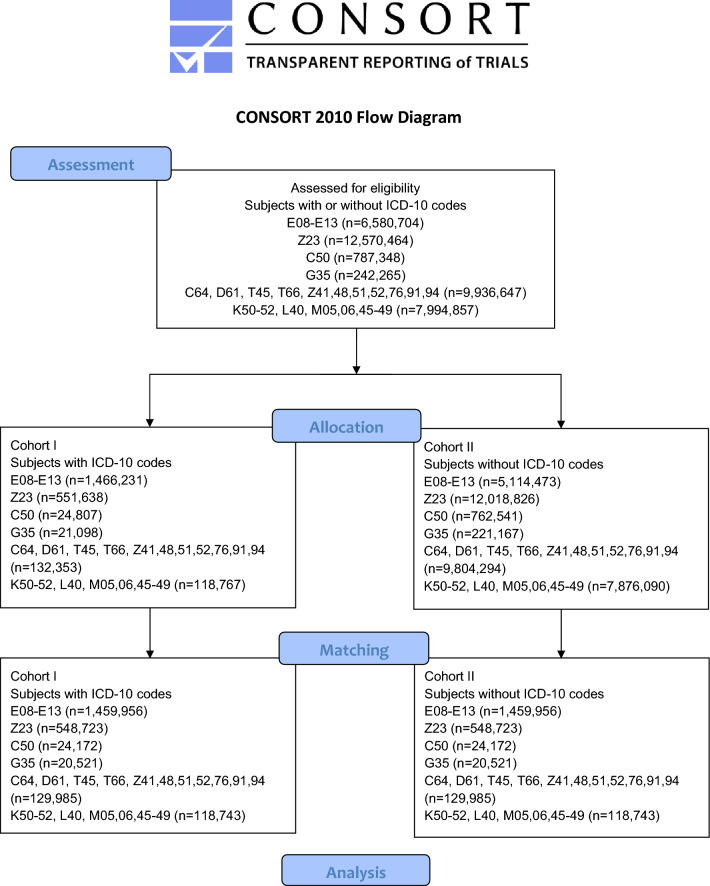
Modified CONSORT flowchart of cohort design and analysis reported in this study.

A total number of 2,303,445 subjects met the inclusion criteria. No individuals had to be excluded for meeting the index event more than 20 years ago. The patient counts before and after propensity score matching of the cohorts are displayed in the Fig. 1 for the respective biologics. Age at index and gender distribution before and after the matching process are also reported in the supplementary Tables S7–S12.

## Results

Using the large-scale nation-wide database of patient de-identified TriNetX EHRs, our study found that there is an increased risk of adverse hepatotoxic events following long-term use of biologics. The current evidence suggests that Insulin glargine is associated with a significant OR of 1.9 (95% CI, 1.8–1.99) causing hepatoxicity in a study population of 1,459,956 patients. The OR with Adalimumab 1.9 (95% CI, 1.72–2.20), Trastuzumab 1.7 (95% CI, 1.2–2.3), Prevnar13 2.3 (95% CI, 2.16–2.60), Pegfilgrastim 2.3 (95% CI, 2.0–2.50), Interferon-Beta1a 1.7 (95% CI, 1.18–2.51) respectively, as shown in Table 1.

**Table 1:** Overall results obtained from the study and the number of patients in cohort.

Biologics	Odds ratio (OR)	95% Cl	Patients in cohort
Adalimumab	1.9	1.72–2.20	124,088
Trastuzumab	1.7	1.2–2.3	24,172
Prevnar13	2.3	2.16–2.60	548,723
Pegfilgrastim	2.3	2.0–2.50	129,985
Interferon-Beta1a	1.7	1.18–2.51	20,521
Insulin glargine	1.9	1.8–1.99	1,459,956

## Discussion

The real-world data obtained through multiple sources which are related to patient health status and behavior in routine clinical practice can give deeper insights into drug safety and efficacy. The RWE -based analysis revealed a statistically significant link between biologics usage and hepatotoxicity in patients. Hepatotoxicity induced by biological agents is a novel emerging cause of drug-induced liver injury (DILI).

In this study, the clinical data in a 1:1 matched‐study design was considered. We have used propensity score‐matched sub‐cohorts to better control for confounding associations that might stem from different distributions of age and gender between the whole dataset and the subset of total patients’ population. We discovered evidence supporting the hepatotoxicity-causing effect of biologics: (i) all biologics considered together had an OR of 1.9 (95% CI, 1.67–2.35), (ii) with Adalimumab 1.9 (95% CI, 1.72–2.20), Trastuzumab 1.7 (95% CI, 1.2–2.3), Prevnar13 2.3 (95% CI, 2.16–2.60), Pegfilgrastim 2.3 (95% CI, 2.0–2.50), Interferon-Beta1a 1.7 (95% CI, 1.18–2.51), and Insulin glargine 1.9 (95% CI, 1.8–1.99).

The causative biologics may induce liver injury via direct different mechanisms triggered by immune dysregulation or indirect molecular events. Hepatic ADRs are being increasingly reported in clinical data and EHRs, and they certainly represent a diagnostic and therapeutic challenge. However, it is prudent to mention that several studies have reported—hepatic injury associated with insulin treatment or overdose, is likely due to glycogenesis rather than inherent liver injury from the insulin usage^14,15^.

In conclusion, our RWE-based study revealed strong evidence for hepatoxic-causing effects of the biologics in largescale multi-organizational cohorts, comprised of long-term biologics users.

Clinicians should be vigilant when using biologics agents and proper monitoring of the safety profile of these therapeutics needs to be considered, as liver damage is a very common event. There is a need to identify and design of novel noninvasive biomarkers to establish the diagnosis of biologics agent-induced DILI, and to monitor prognosis and therapeutic response. Additionally, there is a need for the identification of who patients profile who might experience complete biochemical remissions after drug withdrawal and tolerate retreatment with immunotherapeutic drugs. The clinical monitoring and management of patients treated with biologics should be personalized. RWE studies are important and can play a significant role in providing valuable insights to guide the numerous decisions during the life cycle evidence of drugs to support regulatory decision-making.

## Supplementary Information

Supplementary Information.

## Data Availability

To gain access to the data in the TriNetX research network, a request can be made to TriNetX (https://live.trinetx.com), but costs may be incurred, a data sharing agreement would be necessary, and no patientidentifiable information can be obtained. Data available on reasonable request from the corresponding author.

## Abbreviations

RWE: Real world evidence
RWD: Real world data
HER: Electronic health record
DILI: Drug induced liver injury
OR: Odds ratio
HR: Hazard ratio
ISMS: Information security management system
HIPAA: Health Insurance Portability and Accountability Act
ADRs: Adverse drug reactions
