# Supplementary material for: Using real-world evidence data and digital monitoring to analyze the hepatotoxic profiles of biologics across more than two million patients
Source: Sci Rep. 2023 Jul 5;13:10878. doi: 10.1038/s41598-023-37979-0 (PMC10322819; doi:10.1038/s41598-023-37979-0)
Supplement: Supplementary file 1 — Supplementary Information. [file 41598_2023_37979_MOESM1_ESM.docx]

Supplementary Information

**S1: Cohort 1 and Cohort 2 definition for Adalimumab analysis**

| **Cohort 1 and cohort 2 patient count before and after propensity score matching** | | | |
| --- | --- | --- | --- |
|  | Cohort | Patient count before matching | Patient count after matching |
|  | 1 - adalimumab | 124,110 | 124,088 |
|  | 2 - -adalimumab | 7,831,122 | 124,088 |

| **Cohort 1 (N = 124,110) and cohort 2 (N = 7,831,122) characteristics before propensity score matching** | | | | | | | | | |
| --- | --- | --- | --- | --- | --- | --- | --- | --- | --- |
|  | **Demographics** | | | | | | | | |
|  |  | Cohort | |  | Mean ± SD | Patients | % of Cohort | P-Value | Std diff. |
|  |  | 1 2 | Age | Current Age | 50.6 +/- 17.4 54.3 +/- 23.6 | 124,088 7,506,403 | 100% 100% | <0.001 | 0.180 |
|  |  | 1 2 | AI | Age at Index | 45.9 +/- 17.1 48.3 +/- 24.2 | 124,088 7,506,403 | 100% 100% | <0.001 | 0.114 |
|  |  | 1 2 | F | Female |  | 75,764 4,255,874 | 61.1% 56.7% | <0.001 | 0.089 |
|  |  | 1 2 | M | Male |  | 48,296 3,249,263 | 38.9% 43.3% | <0.001 | 0.089 |
| **Cohort 1 (N = 124,088) and cohort 2 (N = 124,088) characteristics after propensity score matching** | | | | | | | | | |
|  | **Demographics** | | | | | | | | |
|  |  | Cohort | |  | Mean ± SD | Patients | % of Cohort | P-Value | Std diff. |
|  |  | 1 2 | Age | Current Age | 50.6 +/- 17.4 50.6 +/- 17.4 | 124,088 124,088 | 100% 100% | 0.994 | <0.001 |
|  |  | 1 2 | AI | Age at Index | 45.9 +/- 17.1 45.9 +/- 17.1 | 124,088 124,088 | 100% 100% | 0.996 | <0.001 |
|  |  | 1 2 | F | Female |  | 75,764 75,766 | 61.1% 61.1% | 0.993 | <0.001 |
|  |  | 1 2 | M | Male |  | 48,296 48,296 | 38.9% 38.9% | 1 | <0.001 |

**S2: Cohort 1 and Cohort 2 definition for Trastzumab analysis**

| **Cohort 1 and cohort 2 patient count before and after propensity score matching** | | | |
| --- | --- | --- | --- |
|  | Cohort | Patient count before matching | Patient count after matching |
|  | 1 - +trastuzumab | 24,484 | 24,172 |
|  | 2 - -trastuzumab | 757,227 | 24,172 |
|  |  |  |  |

| **Cohort 1 (N = 24,484) and cohort 2 (N = 757,227) characteristics before propensity score matching** | | | | | | | | | |
| --- | --- | --- | --- | --- | --- | --- | --- | --- | --- |
|  | **Demographics** | | | | | | | | |
|  |  | Cohort | |  | Mean ± SD | Patients | % of Cohort | P-Value | Std diff. |
|  |  | 1 2 | AI | Age at Index | 56.6 +/- 13.0 60.8 +/- 14.7 | 24,172 702,391 | 100% 100% | <0.001 | 0.307 |
|  |  | 1 2 | F | Female |  | 23,975 688,383 | 99.2% 98.0% | <0.001 | 0.100 |
|  |  | 1 2 | M | Male |  | 184 13,857 | 0.8% 2.0% | <0.001 | 0.104 |
| **Cohort 1 (N = 24,172) and cohort 2 (N = 24,172) characteristics after propensity score matching** | | | | | | | | | |
|  | **Demographics** | | | | | | | | |
|  |  | Cohort | |  | Mean ± SD | Patients | % of Cohort | P-Value | Std diff. |
|  |  | 1 2 | AI | Age at Index | 56.6 +/- 13.0 56.6 +/- 13.0 | 24,172 24,172 | 100% 100% | 0.994 | <0.001 |
|  |  | 1 2 | F | Female |  | 23,975 23,976 | 99.2% 99.2% | 0.960 | <0.001 |
|  |  | 1 2 | M | Male |  | 184 184 | 0.8% 0.8% | 1 | <0.001 |

**S3: Cohort 1 and Cohort 2 definition for Prevnar13 analysis**

| **Cohort 1 and cohort 2 patient count before and after propensity score matching** | | | |
| --- | --- | --- | --- |
|  | Cohort | Patient count before matching | Patient count after matching |
|  | 1 - +prevnar13 | 551,638 | 548,723 |
|  | 2 - -prevnar13 | 12,018,791 | 548,723 |

| **Cohort 1 (N = 551,638) and cohort 2 (N = 12,018,791) characteristics before propensity score matching** | | | | | | | | | |
| --- | --- | --- | --- | --- | --- | --- | --- | --- | --- |
|  | **Demographics** | | | | | | | | |
|  |  | Cohort | |  | Mean ± SD | Patients | % of Cohort | P-Value | Std diff. |
|  |  | 1 2 | AI | Age at Index | 38.7 +/- 33.1 38.2 +/- 24.5 | 548,723 11,917,011 | 100% 100% | <0.001 | 0.017 |
|  |  | 1 2 | F | Female |  | 285,877 6,655,197 | 52.1% 55.8% | <0.001 | 0.075 |
|  |  | 1 2 | M | Male |  | 262,808 5,258,189 | 47.9% 44.1% | <0.001 | 0.076 |
| **Cohort 1 (N = 548,723) and cohort 2 (N = 548,723) characteristics after propensity score matching** | | | | | | | | | |
|  | **Demographics** | | | | | | | | |
|  |  | Cohort | |  | Mean ± SD | Patients | % of Cohort | P-Value | Std diff. |
|  |  | 1 2 | AI | Age at Index | 38.7 +/- 33.1 38.7 +/- 33.1 | 548,723 548,723 | 100% 100% | 1 | <0.001 |
|  |  | 1 2 | F | Female |  | 285,877 285,877 | 52.1% 52.1% | 1 | <0.001 |
|  |  | 1 2 | M | Male |  | 262,808 262,808 | 47.9% 47.9% | 1 | <0.001 |

**S4: Cohort 1 and Cohort 2 definition for Pegfilgrastim analysis**

| **Cohort 1 and cohort 2 patient count before and after propensity score matching** | | | |
| --- | --- | --- | --- |
|  | Cohort | Patient count before matching | Patient count after matching |
|  | 1 - +pegfilgrastim | 130,096 | 129,985 |
|  | 2 - -pegfilgrastim | 9,742,731 | 129,985 |

| **Cohort 1 (N = 130,096) and cohort 2 (N = 9,742,731) characteristics before propensity score matching** | | | | | | | | | |
| --- | --- | --- | --- | --- | --- | --- | --- | --- | --- |
|  | **Demographics** | | | | | | | | |
|  |  | Cohort | |  | Mean ± SD | Patients | % of Cohort | P-Value | Std diff. |
|  |  | 1 2 | AI | Age at Index | 57.4 +/- 16.8 45.4 +/- 24.6 | 129,985 9,530,975 | 100% 100% | <0.001 | 0.567 |
|  |  | 1 2 | F | Female |  | 78,901 5,171,303 | 60.7% 54.3% | <0.001 | 0.131 |
| **Cohort 1 (N = 129,985) and cohort 2 (N = 129,985) characteristics after propensity score matching** | | | | | | | | | |
|  | **Demographics** | | | | | | | | |
|  |  | Cohort | |  | Mean ± SD | Patients | % of Cohort | P-Value | Std diff. |
|  |  | 1 2 | AI | Age at Index | 57.4 +/- 16.8 57.4 +/- 16.8 | 129,985 129,985 | 100% 100% | 1 | <0.001 |
|  |  | 1 2 | F | Female |  | 78,901 78,901 | 60.7% 60.7% | 1 | <0.001 |

**S5: Cohort 1 and Cohort 2 definition for Interferon-Beta1a analysis**

| **Cohort 1 and cohort 2 patient count before and after propensity score matching** | | | |
| --- | --- | --- | --- |
|  | Cohort | Patient count before matching | Patient count after matching |
|  | 1 - +interferonBeta1a | 21,097 | 20,521 |
|  | 2 - -interferonBeta1a | 220,516 | 20,521 |

| **Cohort 1 (N = 21,097) and cohort 2 (N = 220,516) characteristics before propensity score matching** | | | | | | | | | |
| --- | --- | --- | --- | --- | --- | --- | --- | --- | --- |
|  | **Demographics** | | | | | | | | |
|  |  | Cohort | |  | Mean ± SD | Patients | % of Cohort | P-Value | Std diff. |
|  |  | 1 2 | AI | Age at Index | 47.5 +/- 13.0 48.6 +/- 15.1 | 20,521 211,784 | 100% 100% | <0.001 | 0.075 |
|  |  | 1 2 | F | Female |  | 15,589 157,068 | 76.0% 74.2% | <0.001 | 0.042 |
| **Cohort 1 (N = 20,521) and cohort 2 (N = 20,521) characteristics after propensity score matching** | | | | | | | | | |
|  | **Demographics** | | | | | | | | |
|  |  | Cohort | |  | Mean ± SD | Patients | % of Cohort | P-Value | Std diff. |
|  |  | 1 2 | AI | Age at Index | 47.5 +/- 13.0 47.5 +/- 13.0 | 20,521 20,521 | 100% 100% | 1 | <0.001 |
|  |  | 1 2 | F | Female |  | 15,589 15,589 | 76.0% 76.0% | 1 | <0.001 |

**S6: Cohort 1 and Cohort 2 definition for Insulin Glargine analysis**

| **Cohort 1 and cohort 2 patient count before and after propensity score matching** | | | |
| --- | --- | --- | --- |
|  | Cohort | Patient count before matching | Patient count after matching |
|  | 1 - +insulinGlargine | 1,461,230 | 1,459,956 |
|  | 2 - -insulinGlargine | 5,089,075 | 1,459,956 |

| **Cohort 1 (N = 1,461,230) and cohort 2 (N = 5,089,075) characteristics before propensity score matching** | | | | | | | | | |
| --- | --- | --- | --- | --- | --- | --- | --- | --- | --- |
|  | **Demographics** | | | | | | | | |
|  |  | Cohort | |  | Mean ± SD | Patients | % of Cohort | P-Value | Std diff. |
|  |  | 1 2 | AI | Age at Index | 57.9 +/- 17.8 58.3 +/- 17.8 | 1,459,956 4,945,135 | 100% 100% | <0.001 | 0.020 |
|  |  | 1 2 | F | Female |  | 693,132 2,521,519 | 47.5% 51.0% | <0.001 | 0.070 |
|  |  | 1 2 | M | Male |  | 766,629 2,421,522 | 52.5% 49.0% | <0.001 | 0.071 |
| **Cohort 1 (N = 1,459,956) and cohort 2 (N = 1,459,956) characteristics after propensity score matching** | | | | | | | | | |
|  | **Demographics** | | | | | | | | |
|  |  | Cohort | |  | Mean ± SD | Patients | % of Cohort | P-Value | Std diff. |
|  |  | 1 2 | AI | Age at Index | 57.9 +/- 17.8 57.9 +/- 17.8 | 1,459,956 1,459,956 | 100% 100% | 1.000 | <0.001 |
|  |  | 1 2 | F | Female |  | 693,132 693,132 | 47.5% 47.5% | 1 | <0.001 |
|  |  | 1 2 | M | Male |  | 766,629 766,629 | 52.5% 52.5% | 1 | <0.001 |

**S7: Adalimumab hepatoxicity outcomes**

| **1 hepatotoxicity** | | | | | | | | | | | | | | | |
| --- | --- | --- | --- | --- | --- | --- | --- | --- | --- | --- | --- | --- | --- | --- | --- |
| **Risk analysis** | | | | | | | | | | | | | |  |  |
|  | | Cohort | | | Patients in cohort | | Patients with outcome | | Risk | | | | | |  |
|  |  | | 1 | adalimumab | | 124,088 | | 574 | | 0.005 | | | | | |
|  |  | | 2 | -adalimumab | | 124,088 | | 290 | | 0.002 | | | | | |
|  | | | | | | | | | | | | | | | |
|  |  | |  | | |  | | 95% CI | | z | p |  |  |  |  |
|  |  | | **Risk Difference** | | | 0.002 | | (0.002, 0.003) | | 9.679 | 0.000 |  |  |  |  |
|  |  | | **Risk Ratio** | | | 1.979 | | (1.719, 2.279) | | N/A | N/A |  |  |  |  |
|  |  | | **Odds Ratio** | | | 1.984 | | (1.722, 2.285) | | N/A | N/A |  |  |  |  |

| **Kaplan - Meier survival analysis** | | | | | | | | |  |
| --- | --- | --- | --- | --- | --- | --- | --- | --- | --- |
| Cohort | | Patients in cohort | Patients with outcome | Median survival (days) | Survival probability at end of time window | | |  |  |
| 1 | adalimumab | 124,088 | 574 | -- | 99.27% | | |  |  |
| 2 | -adalimumab | 124,088 | 290 | -- | 99.60% | | |  |  |
|  | | | | | | | | | |
|  | | χ^2^ | df | p |  |  |  |  |  |
| **Log-Rank Test** | | 50.775 | 1 | 0.000 |  |  |  |  |  |
|  | | | | | | | | | |
|  | | Hazard Ratio | 95% CI | χ^2^ | df | p | |  |  |
| **Hazard Ratio and Proportionality** | | 1.662 | (1.443, 1.914) | 8.538 | 1 | 0.003 | |  |  |

**S8: Trastuzumab hepatoxicity outcomes**

| **1 hepatotoxicity** | | | | | | | | | | | | | | | |
| --- | --- | --- | --- | --- | --- | --- | --- | --- | --- | --- | --- | --- | --- | --- | --- |
| **Risk analysis** | | | | | | | | | | | | | |  |  |
|  | | Cohort | | | Patients in cohort | | Patients with outcome | | Risk | | | | | |  |
|  |  | | 1 | +trastuzumab | | 24,172 | | 105 | | 0.004 | | | | | |
|  |  | | 2 | -trastuzumab | | 24,172 | | 61 | | 0.003 | | | | | |
|  | | | | | | | | | | | | | | | |
|  |  | |  | | |  | | 95% CI | | z | p |  |  | | |
|  |  | | **Risk Difference** | | | 0.002 | | (0.001, 0.003) | | 3.421 | 0.001 |  |  | | |
|  |  | | **Risk Ratio** | | | 1.721 | | (1.256, 2.359) | | N/A | N/A |  |  | | |
|  |  | | **Odds Ratio** | | | 1.724 | | (1.257, 2.365) | | N/A | N/A |  |  | | |

| **Kaplan - Meier survival analysis** | | | | | | | | | |  |
| --- | --- | --- | --- | --- | --- | --- | --- | --- | --- | --- |
|  |  | Cohort | | Patients in cohort | Patients with outcome | Median survival (days) | Survival probability at end of time window | | | |
|  |  | 1 | +trastuzumab | 24,172 | 105 | -- | 99.11% | | | |
|  |  | 2 | -trastuzumab | 24,172 | 61 | -- | 98.97% | | | |
|  | | | | | | | | | | |
|  |  |  | | χ^2^ | df | p |  |  |  | |
|  |  | **Log-Rank Test** | | 10.076 | 1 | 0.002 |  |  |  | |
|  | | | | | | | | | | |
|  |  |  | | Hazard Ratio | 95% CI | χ^2^ | df | p | | |
|  |  | **Hazard Ratio and Proportionality** | | 1.660 | (1.210, 2.277) | 0.176 | 1 | 0.675 | | |

**S9: Prevnar13 hepatoxicity outcomes**

| **1 hepatotoxicity** | | | | | | | | | | | | | | | |
| --- | --- | --- | --- | --- | --- | --- | --- | --- | --- | --- | --- | --- | --- | --- | --- |
| **Risk analysis** | | | | | | | | | | | | | |  |  |
|  | | Cohort | | | Patients in cohort | | Patients with outcome | | Risk | | | | | |  |
|  |  | | 1 | +prevnar13 | | 548,723 | | 1,527 | | 0.003 | | | | | |
|  |  | | 2 | -prevnar13 | | 548,723 | | 645 | | 0.001 | | | | | |
|  | | | | | | | | | | | | | | | |
|  |  | |  | | |  | | 95% CI | | z | p |  |  | | |
|  |  | | **Risk Difference** | | | 0.002 | | (0.001, 0.002) | | 18.944 | 0.000 |  |  | | |
|  |  | | **Risk Ratio** | | | 2.367 | | (2.159, 2.595) | | N/A | N/A |  |  | | |
|  |  | | **Odds Ratio** | | | 2.371 | | (2.163, 2.600) | | N/A | N/A |  |  | | |

|  | **Kaplan - Meier survival analysis** | | | | | | | | | |
| --- | --- | --- | --- | --- | --- | --- | --- | --- | --- | --- |
|  | |  | Cohort | | Patients in cohort | Patients with outcome | Median survival (days) | Survival probability at end of time window | | |
|  | |  | 1 | +prevnar13 | 548,723 | 1,527 | -- | 99.45% | | |
|  | |  | 2 | -prevnar13 | 548,723 | 645 | -- | 99.47% | | |
|  | | | | | | | | | | |
|  | |  |  | | χ^2^ | df | p |  |  |  |
|  | |  | **Log-Rank Test** | | 252.700 | 1 | 0.000 |  |  |  |
|  | | | | | | | | | | |
|  | |  |  | | Hazard Ratio | 95% CI | χ^2^ | df | p | |
|  | |  | **Hazard Ratio and Proportionality** | | 2.077 | (1.894, 2.277) | 32.578 | 1 | 0.000 | |

**S10: Pegfilgrastim hepatoxicity outcomes**

| **1 hepatoxicity** | | | | | | | | | | | | | | | |
| --- | --- | --- | --- | --- | --- | --- | --- | --- | --- | --- | --- | --- | --- | --- | --- |
| **Risk analysis** | | | | | | | | | | | | | |  |  |
|  | | Cohort | | | Patients in cohort | | Patients with outcome | | Risk | | | | | |  |
|  |  | | 1 | +pegfilgrastim | | 129,985 | | 981 | | 0.008 | | | | | |
|  |  | | 2 | -pegfilgrastim | | 129,985 | | 440 | | 0.003 | | | | | |
|  | | | | | | | | | | | | | | | |
|  |  | |  | | |  | | 95% CI | | z | p |  |  |  |  |
|  |  | | **Risk Difference** | | | 0.004 | | (0.004, 0.005) | | 14.391 | 0.000 |  |  |  |  |
|  |  | | **Risk Ratio** | | | 2.230 | | (1.993, 2.494) | | N/A | N/A |  |  |  |  |
|  |  | | **Odds Ratio** | | | 2.239 | | (2.000, 2.506) | | N/A | N/A |  |  |  |  |

| **Kaplan - Meier survival analysis** | | | | | | | | |  |
| --- | --- | --- | --- | --- | --- | --- | --- | --- | --- |
| Cohort | | Patients in cohort | Patients with outcome | Median survival (days) | Survival probability at end of time window | | |  |  |
| 1 | +pegfilgrastim | 129,985 | 981 | -- | 98.10% | | |  |  |
| 2 | -pegfilgrastim | 129,985 | 440 | -- | 98.99% | | |  |  |
|  | | | | | | | | | |
|  | | χ^2^ | df | p |  |  |  |  |  |
| **Log-Rank Test** | | 206.219 | 1 | 0.000 |  |  |  |  |  |
|  | | | | | | | | | |
|  | | Hazard Ratio | 95% CI | χ^2^ | df | p | |  |  |
| **Hazard Ratio and Proportionality** | | 2.233 | (1.995, 2.500) | 0.000 | 1 | 0.987 | |  |  |

**S11: Interferon-Beta1a hepatoxicity outcomes**

| **1 hepatotoxicity** | | | | | | | | | | | | | | | |
| --- | --- | --- | --- | --- | --- | --- | --- | --- | --- | --- | --- | --- | --- | --- | --- |
| **Risk analysis** | | | | | | | | | | | | | |  |  |
|  | | Cohort | | | Patients in cohort | | Patients with outcome | | Risk | | | | | |  |
|  |  | | 1 | +interferonBeta1a | | 20,521 | | 74 | | 0.004 | | | | | |
|  |  | | 2 | -interferonBeta1a | | 20,521 | | 43 | | 0.002 | | | | | |
|  | | | | | | | | | | | | | | | |
|  |  | |  | | |  | | 95% CI | | z | p |  |  |  |  |
|  |  | | **Risk Difference** | | | 0.002 | | (0.000, 0.003) | | 2.870 | 0.004 |  |  |  |  |
|  |  | | **Risk Ratio** | | | 1.721 | | (1.182, 2.505) | | N/A | N/A |  |  |  |  |
|  |  | | **Odds Ratio** | | | 1.724 | | (1.183, 2.511) | | N/A | N/A |  |  |  |  |

| **Kaplan - Meier survival analysis** | | | | | | | | |  |
| --- | --- | --- | --- | --- | --- | --- | --- | --- | --- |
| Cohort | | Patients in cohort | Patients with outcome | Median survival (days) | Survival probability at end of time window | | |  |  |
| 1 | +interferonBeta1a | 20,521 | 74 | -- | 99.32% | | |  |  |
| 2 | -interferonBeta1a | 20,521 | 43 | -- | 99.52% | | |  |  |
|  | | | | | | | | | |
|  | | χ^2^ | df | p |  |  |  |  |  |
| **Log-Rank Test** | | 1.313 | 1 | 0.252 |  |  |  |  |  |
|  | | | | | | | | | |
|  | | Hazard Ratio | 95% CI | χ^2^ | df | p | |  |  |
| **Hazard Ratio and Proportionality** | | 1.247 | (0.854, 1.819) | 1.354 | 1 | 0.245 | |  |  |

**S12: Insulin Glargine hepatoxicity outcomes**

| **1 hepatotoxicity** | | | | | | | | | | | | | | | |
| --- | --- | --- | --- | --- | --- | --- | --- | --- | --- | --- | --- | --- | --- | --- | --- |
| **Risk analysis** | | | | | | | | | | | | | |  |  |
|  | | Cohort | | | Patients in cohort | | Patients with outcome | | Risk | | | | | |  |
|  |  | | 1 | +insulinGlargine | | 1,459,956 | | 6,144 | | 0.004 | | | | | |
|  |  | | 2 | -insulinGlargine | | 1,459,956 | | 3,217 | | 0.002 | | | | | |
|  | | | | | | | | | | | | | | | |
|  |  | |  | | |  | | 95% CI | | z | p |  |  | | |
|  |  | | **Risk Difference** | | | 0.002 | | (0.002, 0.002) | | 30.301 | 0.000 |  |  | | |
|  |  | | **Risk Ratio** | | | 1.910 | | (1.830, 1.993) | | N/A | N/A |  |  | | |
|  |  | | **Odds Ratio** | | | 1.914 | | (1.834, 1.997) | | N/A | N/A |  |  | | |

| **Kaplan - Meier survival analysis** | | | | | | | | |  |
| --- | --- | --- | --- | --- | --- | --- | --- | --- | --- |
| Cohort | | Patients in cohort | Patients with outcome | Median survival (days) | Survival probability at end of time window | | |  |  |
| 1 | +insulinGlargine | 1,459,956 | 6,144 | -- | 98.66% | | |  |  |
| 2 | -insulinGlargine | 1,459,956 | 3,217 | -- | 99.27% | | |  |  |
|  | | | | | | | | | |
|  | | χ^2^ | df | p |  |  |  |  |  |
| **Log-Rank Test** | | 908.134 | 1 | 0.000 |  |  |  |  |  |
|  | | | | | | | | | |
|  | | Hazard Ratio | 95% CI | χ^2^ | df | p | |  |  |
| **Hazard Ratio and Proportionality** | | 1.906 | (1.827, 1.989) | 0.255 | 1 | 0.613 | |  |  |
